# Southern Dental Association

**Published:** 1888-01

**Authors:** 


AMERICAN JOURNAL

—OF—

DENTAL SCIENCE.

VOL. XXI. THIRD SERIES—JANUARY, 1888. No. 9.

ARTICLE I.

### SOUTHERN DENTAL ASSOCIATION, OLD POINT COMFORT, VA.—NINETEENTH ANNUAL SESSION.

REPORTED BY "MRS. M. W. J."

Dr. J. B. Hodgkin said that without statistics or rhetoric, he had observed that constitutions were laid out on certain lines. His father, at 82, had perfect teeth, and yet he used no tooth wash, and had a holy horror of dentists.

One man has a long nose and short legs, another has a turned up nose and very long legs; one has light, straight hair, another has dark, curly hair; when the lines were laid down that made him that way he got the tread or type, not only of his legs, and his hair, but also of his eyes, and his teeth. When were those lines laid down? Some one asks, "when shall I begin to educate my child?" It was begun two hundred years ago! A mother brings her child to me:

to examine her teeth. I can only say to her. "I will do the best I can for her, but she will yet wear artificial teeth." When the lines of the constitution were laid down, the teeth gôt a certain stamp, and, and you cannot stamp it out.

(Repeated cries for Atkinson! Atkinson!)

Dr. W. H. Atkinson took the stand saying: "There must have been many stamps at work in the molecular construction of that call for Atkinson!" He said that he had warm words of greeting for his brethren in the South, whom he loved because they loved the truth, and manifested such earnest wishes to attain to it.

The forces that move men, and stamp out their career, were at work before there were any inhabitants on this planet. These forces were transmitted down through the ages, from the mother to the child, through the ministration of the male parent.

In the discussions, he had been pained to see how many propositions had been started on mere assumptions, without any solid ground or foundation, except that of "stamps," and even that was not defined so that one could get hold of it clearly. A knowledge of embryology is essential to the understanding of the question involved. Each one should walk in obedience to his own highest conception of truth, taking the step that the light within him shows it to be the proper step for him to take. Words fresh from hearts warmed and illuminated with divine truth are better than memorized trash in books; but don't take away the gods of the past till you have something better to put in their places. What is the use of the teeth? to comminute food; the babe has no teeth because the nutriment in milk has been comminuted and it holds stored radiance. With our present habits of civilization we have but little real need of teeth, our food is so prepared for us. When the natural mode of feeding was in vogue the food kept the teeth in order. The highest duty of the professional man is to so teach the subject he deals with that he shall no longer need his ministrations. We should not fear to trust the inspiration of the

Almighty that giveth us understanding, but should live constantly in the illumination that comes from without.

Dr. Freidrichs (New Orleans) thought that though the original stamp or tread might be about the same in two individuals—whether of the human race or seeds of plants—we had the power of control over their development into mere different finalities; of the two human beings, one might be starved and neglected, the other well fed, sheltered and nursed; of the two seeds, one might be planted in rich soil and have no care, the other planted in rich soil and have every attention; the results would be very different in each case, notwithstanding the original stamp or trend.

Dr. A. W. Harlan (Chicago) being introduced, stated that at the meeting of the American Dental Association, a committee had been appointed to confer with a similar committee from the Southern Association with reference to the acceptance by the latter of an invitation to hold the next annual meeting of the two Associations in joint session. He therefore asked the appointment of such a committee that the conference might be held.

Dr. J. Rollo Knapp (New Orleans) made the motion and asked that a committee of five be at once appointed by the President.

Dr. W. H. Richards (Knoxville, Tenn.,) moved to amend and postpone the appointment until a more convenient hour, as it was then nearing midnight.

A motion made to adjourn was negatived.

The President said he would have preferred time for consideration, that he might make a judicious appointment, but under the stress of circumstances, he yielded, and named Dr's J. R. Knapp. E. S. Chisholm, Winkler and Storey; Dr's. Harlan, Waters, McElhaney, Shepherd and Wardlow being the Committee from the American Association.

Adjourned.

Friday, September 2nd.

Called to order 10:30 a. m. The President in the chair.

A telegram was read from the President of the American Dental Association, congratulating the Southern Association in its successful meeting, and expressing regrets at inability to be present, owing to illness of his wife.

Prof. Hubbard, of the Mehary College was introduced, who addressed the Association on the subject of his college for colored youths, and the gratifying progress made during the past year in the dental department.

Prof. J. B. Hodgkin then read the following report from the committee appointed to draft resolutions in memory of the late Dr. J. R. Walker, of New Orleans, La.

Dr. J. R. Walker was born in N. Y. State, in 1830; was educated in Ohio; studied dentistry in the private offices of several dentists; settled in New Orleans in 1854, where he practiced continuously (except during his service in the Confederate army). He was twice Vice-President of the American Dental Association, President of the Southern Dental Association, and was President elect of the National Dental Association at the time of his death.

He had been a Fellow of the American Association for the advancement of science, and Vice-President of the New Orleans Academy of Science, and a frequent contributor to scientific and dental journals.

He died at his summer home in Bay St. Louis, Miss., June 22nd, 1887, leaving a widow and five children.

Such was the man, who, were he still alive, would stand among us to day. But, to read his career as we should, we must needs go to the unwritten records made on our minds and hearts by the man himself.

Strong, magnetic, attractive, and, above all, self-sacrificing as a worker for tne profession he loved so dearly, and for this association, which was his pride. One of the very first in conceiving the idea of its organization, and one of the foremost in working for its early life, he spared no pains and hesitated at no self-sacrifice which would forward the interests of the Southern Dental Association. His time was its time, his money its money, his thought and care were the thought and care of the organization which, frowned at by some and sneered at by others, kept alive because a few choice spirits, of whom he was one, resolved that it should not die. He was with it when in its early days it met in the Middle West, so feeble that the dentists of the city in which it assembled had to elect some of the local practitioners members before a quorum could be had. And he was with it when it stood strong without help from other societies.

Would that he were here to see the full fruition of his hopes and labors in the nearly 350 assembled here to-day!

But he is gone! We still hear, in imagination, his voice in debate and counsel; we see the kindly glance of the eye, and hear the cherry tone of the voice of the always good fellow. No differences of sentiment made him less a friend; no clashing of interests made him less enthusiastic for his calling. So strong and manly, yet so genial and kindly, that even those he fought loved him.

It is a trite saying that "He who makes two blades of grass grow where but one grew before, is a benefactor of his race," but we hold that he who tries to make one more grow, even if he fails in his endeavor, is no less a benefactor in motive and in heart. And so, if it were that no association lived to "rise up and call him blessed" as one of its fathers; had this Southern perished by frost or blight, yet could we well applaud Dr. J. R. Walker and say, "He hath done what he could."

The motive makes the man, his impulses, his character, his heart; and we reverence that, though no fruition come of all its hopes and aspirations. The "Well done, good and faithful servant" was spoken of him who was faithful over a few things.

Our friend had his convictions, and the courage to sustain them. We might not always agree with him, but they worked his individuality, and made for him a character. Enemies may snarl, but the man is there. The lesson of his life, to us, is worth learning.

He had a purpose, and tried to make circumstances aid his purpose rather than allow circumstances to mould his purpose.

We must ponder this lesson. Purpose! Circumstance! these two. Shall we drift with the tide, or take advantage of it? Circumstance without purpose is a tide to float on to destruction. Purpose, with circumstance. takes whithersoever we will.

*Resolved*, That this association express its deep regret at the death of its late fellow-worker, Dr. J. Walker; its profound sympathy for his widow and children, and a determination that the fruits of the seed he planted with us shall have a rich harvest in the prosperity of this association.

*Resolved*, That a page of the minute book be inscribed to his memory, and that a copy of these resolutions be sent to his widow and children.

On motion of Dr. Catching the resolutions were ordered spread on the minutes, and a copy furnished the family of the deceased.

The following report from the "Committee of Conference" was read and accepted. [No report furnished.–Ed.]

After some announcements from Dr. J. Hall Moore, as to excursions, etc., a vote of thanks was tendered the Virginia State Association for its generous hospitality in this and other respects.

Thanks were also voted to the S. S. White Co., and Mr. J. W. Selby for assistance in arranging for clinics. etc.

On motion of Dr. Chisholm, thanks were tendered the Hygiea Hotel, and the Railroads for reduced rates.

The President then introduced Dr. A. M. Lulie, a veteran with bleached locks, who had devoted the talents and energies of a lifetime to the advancement of the dental profession, and the originator and inventor of cohesive gold foil.

Dr. Lulie then read a paper, giving the reasons why he had been deemed worthy of this distinction.

The discussion of Dental Hygiene was then continued.

"Mrs. M. W. J." the author of "Letters to Mothers on the care of Children's Teeth," being called upon, stated briefly the practical results of systematic cleanliness and diet in overcoming both heredity and environment in the children of a number of families, and especially her own five children. In the latter case, from their ancestry on both sides they were eminently liable to inherit very poor teeth, increased by residence in a portion of country unusually destitute of mineral elements in drinking water, etc.—Through the negligence and skepticism of the mother, no special precautions were adopted with the oldest child, until the age of three years, when the teeth being very soft and decaying rapidly, thirteen fillings were inserted, and a radical change instituted in care and diet; with the gratifying result of so hardening the deciduous teeth that no fillings were needed after the third birthday, and all the teeth but one retained until shed by nature's process. The second denture is unusually fine in structure and regularity, and at the age of nearly twenty-one, the teeth are all in place, in well developed, regular arches, without any fillings except that the imperfect fissures of the first molars were early cut out and filled to prevent decay. The second child retained all the deciduous teeth in perfect condition, without one exception. He erupted a central incisor with a pin-head-sized spot of yellow, softened dentine, on the labial surface, midway between cutting edge and gum which melted out, and at the of *eleven months*, was filled with cement. At the age of two years it was refilled with amalgam, and at the age of three years with gold—more as a curiosity than from any real necesity. The permanent incisor was similarly marked in the same position with a white spot, which, without softening, has, at the age of eighteen, grown down to the cutting edge of the tooth, like a white spot on a finger nail. As the result of special dental hygiene, the five children (the youngest now eleven years old) have all exceptionally fine dentures. The third child, however, presents the anomaly of having never erupted the second bicuspids, nor the left superior lateral and canine. The deciduous teeth, when shed were absolutely perfect, without a speck of decay, and with about the usual amount of absorption.

Dr. Dunlap (Selma, Ala.,) read a communication from the Alabama State Dental Society, on the subject of lectures to the public on Dental Hygiene.

On motion, the paper was adopted, and laid over for action next year.

Dr. Richards (Knoxville, Tenn.,) spoke of the lamentable condition of the teeth of the common soldiers in Fortress Monroe, as evidenced by the patients furnished for clinics, and said that something should be done to direct the attention of Government to this matter. The appointment of dentists in the army and navy would not interfere with the work of physicians and surgeons. They should co-operate with each other. A noted physician had said the army could be fed at one-third less expense if the soldiers had good molars. He hoped the Association would take some action in this matter.

The subject of Pathology and Therapeutics was called.

Dr. W. C. Wardlaw (Augusta, Ga.,) read a paper entitled,

NEURALGIA, ITS ASSOCIATION WITH DENTAL LESION.

Dr. J. J. R. Patrick then read a paper on the correction of Irregularities, illustrated by a large working-model and a series of charts.

Dr. W. H. Atkinson criticised the term "exuviation" to designate the absorption of the deciduous teeth. He said there was a return to embryonic conditions at the point where absorption takes place, and the debris taken up at the point where the molecular metamorphorsis occurs. He would also like to correct all the fanciful generalizations offered in the explanations, but time would not permit.

Dr. Patrick said that "absorption" was an indefinite term which did not mean anything in particular. There were two kinds of absorption, external and internal, one of construction, one of destruction; one builds up, the other tears down; while we are arbsorbing that which lower organisms have built up, the earth and the atmosphere around us are absorbing our tissues. Cavier uses exuviation as the correct scientific physiological term. It is a physiological process; opposed to the pathological process exfoliation.

Dr. Atkinson said this was mere discrimination without distinction. Exuviation was throwing off a cornuous layer of epithelium, made possible by taking fluids from the external walls of the cells a process of throwing off through desication. The lime-salts are melted and carried away through the lymphatics.

The hour having arrived for the election of officers, Dr. J. R. Knapp, (New Orleans,) said that as the Association had decided to go to Kentucky next year, it would be most fitting to have a standard-bearer from that State. He therefore nominated, Dr. J. H. Prewitt, of Madisonville, Ky., the Demosthenes of American Dentistry.

The nominations was recorded by Dr. Storey (Dallas, Texas.)

Dr. Freidrichs (New Orleans,) moved to close the nominations.

This was not seconded, and Dr. Winkler (Augusta, Ga.,) nominated Dr. B. H. Catching, First Vice-President of the Association one who has labored for years to advance the interests of the profession, and who had contributed largely towards building the Southern Association up to its present status. On a previous occasion he had withdrawn from the contest when there was every probability that he would have been elected and it was only justice that he should have the honor now.

The nomination was seconded and nominations closed.

Five minutes' recess was taken to enable newly elected members to pay their dues, Dr. J. R. Knapp called attention to the fact that payment of the initiation fee counts as the first year's dues, and entitles to vote.

Drs. Winkler and J. R. Knapp were appointed tellers.

On the first ballot Dr. B. H. Catching receiving a majority of all the votes cast, his election was, on motion of Dr. Prewitt, declared unanimous.

The President said that the result afforded him great satisfaction, as the newly elected President was a man tried and true; one in whom all confidence would be placed, and one who would know how to fulfill every duty. He said that he was about to congratulate Dr. Catching, but instead he would congratulate the Southern Association on the wisdom of their choice.

Dr. Catching replied briefly, accepting the honor with sincere appreciation of all that it implied, and declaring that he would, if possible, work harder in the future than he had done in the past. He said that he wished that he had the gift of oratory of his friend from Kentucky, that he might thank them in fitting terms for honor conferred.

Dr. Winkler then nominated Dr. J. H. Prewitt for First Vice-President, and the Secretary was instructed to cast the ballot.

Dr. Prewitt, in accepting the position, said that he had been honored beyond his merits. He promised that he would try, in meeting in his own State, to play a correct Pythias to Catching's Damon.

Dr. W. N. Morrison, St. Louis, Mo., was elected Second Vice-President.

Dr. J. Hall Moore, Richmond, Va. Third Vice-President.

Dr. Thackston expressed his gratification at the election of this old and tried friend, to whose labor it was due that the present meeting had been the great success it was. His past services were the guarantee of what the future would be.

Dr. J. Y. Crawford, Nashville, Tenn., Corresponding Secretary.

Dr. L. P. Dotterer, Charleston, S. C., Recording Secretary.

Dr. H. A. Lowrance, Athens, Ga., Treasurer.

Were all re-elected to their several offices.

Drs. Edwards and Doyle, of Louisville, Ky.; and Dr. Wm. Dancy, of Jacksonville, Fla.; were elected on the Executive Committee.

Adjourned to 8:30 p. m.

---

FRIDAY, SEPTEMBER 2, 9 P. M.

The President in the chair.

Dr. McKellops, chairman of the committee on Voluntary Essays, stated that during his last illness, Dr. J. R. Walker, of New Orleans, had prepared a paper for the present meeting, which had been given to the committee.

On motion the paper was read by title:

THE FUTURE OF DENTISTRY—A PROPHECY, and ordered published in the proceedings.

Dr. W. D. Dunlap stated that Dr. Reese, of Galveston, Texas, had promised a paper if his health permitted, but that the paper had not come to hand.

The President replied that he would be pleased to receive it, and would order it published.

The subject of Pathology and Therapeutics, and especially the paper from Dr. Wardlaw, being open to discussion.

Dr. Genese (Baltimore) gave the history of a case of so-called neuralgia, due to dental lesion. The lady had been under treatment of physicians for some time, for neuralgia; five good teeth had also been sacrificed, without relief. The pain was severe at night, with intermittent fever, loss of appetite, and great depression of spirits. Her remaining teeth were apparently good and of sound structure. As a sedative employed one not used here—the solid extract of *Papaner Alba*, spread on the margin of the gums an hour before seeking sleep (with the precaution given to swallow as little as possible.) As the effect she had the best night's sleep she had enjoyed for three or four months. Thorough examination, the next day, revealed softened dentine between the bicuspids all around a filling, which had exposed the pulp. Capping the pulp and a plastic filling gave entire relief, and there has been no recurrence of the pain—now six weeks since the operation.

Dr. R. Finley Hunt said that though he agreed in the main with the paper, he must differ from Dr. Wardlaw on some points, especially in his positive statement that neuralgia, as a rule, is produced by lesions of the dental structure. Pure neuralgia exists without any special lesion, being a systemic abnormal condition. The pain due to dental lesions is not pure neuralgia, which may exist when the teeth are perfect—without a particle of decay. There is much neuralgic pain due to defective teeth, and to deposits of even a very small quantity of salivary calculus; another form is due to too incessant smoking, and is cured by limiting the number of cigars. We are continually encountering the difficulty of indefinite nomenclature. We should endeavor to fix upon terms that when we use them to-day shall mean the same thing to-morrow.

Dr. L. G. Noel (Nashville, Tenn.,) read a paper entitled,

"ETEOLOGY OF CARIES OF THE TEETH VIEWED FROM THE STANDPOINT OF PHYSIOLOGICAL CHEMISTRY."

The paper was passed without discussion, and Prof. Hodgkin (Baltimore), read a short paper, entitled,

"ARE WE JUSTIFIED IN PROMISING SUCCESS IN RE-PLANTATION?"

Dr. Winkler then requested Dr. Morgan to explain the experiment made with cohesive gold.

Dr. Morgan said that the question had been raised whether aqua ammonia would render cohesive foil non-cohesive, and the matter had been put to the test as follows.

A bat of cotton was laid in the bottom of a box, over which a network of silk floss was stretched, on one end of which he had placed a sheet of cohesive gold "1,000 fine," No. 5, which he had examined and found decidedly cohesive. On the other end of the netting Dr. Winkler laid a sheet of No. 6 "globe foil, extra cohesive." The cotton was then saturated with ammonia, the cover closed and weighted and left closed for two hours. When the box was opened it was found that the "1,000 fine" had lost its cohesion entirely. Of the other, one side of gold was still cohesive, but not enough to make it tear after being pressed together in paper. He therefore claimed that the question was settled in the affirmative.

Dr. Winkler said that Dr. Morgan had stated the facts very clearly, but that he did not accept the experiment as entirely satisfactory. The box was narrow, the amount of cotton was considerable, the fibres being raised up by the absorption of ammonia. One oz. ammonia was used in the box. The box should have been opened sooner, the moisture from the evaporation of the water of the aqua ammonia having had time to take effect on the gold. The foil was not very cohesive, and the upper surface was as much so after as before the experiment. The gold should have been freshly annealed. The cover had sprung; the box was wet, enough water remaining to be poured out of the box. Only one sheet was found that was not at all cohesive, and that was like it had been put in a water bath; in fact, he thought the effect produced due entirely to moisture, and not to the fumes of ammonia.

Dr. Morgan said he had not made any claim for the "fumes of ammonia;" he had said "aqua ammonia," and did not profess to know to what the effect was due. He would admit, however, that the time required had been longer than he had anticipated. He had often done it in five minutes, by passing the foil over the mouth of a vial of aqua ammonia. He had consulted various metallurgical authorities—among them Dr. Leslie and Dr. J. J. R. Patrick, and they said, "Oh, yes; it would do it beyond question."

Dr. Winkler asked why he supposed one side was not affected?

Dr. Morgan replied, because the fumes were stronger on the under side, the gold should have been turned over.

Dr. Beach (Clarkesville, Tenn.) said the only thing proved was that *something* had caused the change; whether water or ammonia was not proved. Cohesive foil can be made non-cohesive by dipping in water and drying, but it cannot be relied upon with certainty. Chloroform will also produce the same effect.

Prof. Hodgkin moved that the subject be passed, and the experiments continued at home.

On motion of Dr. Hunt, Drs. Morgan and Winkler were appointed a committee to pursue the experiment and report at the next meeting.

The President suggested that the whole thing pertained to metallurgy rather than to the topics properly under discussion.

Dr. Morrison (St. Louis) said that from his long experience in replantation, he wished to answer Dr. Hodgkins' paper. He had had so many successful cases that he considered it in every way a legitimate operation. The case of Dr. Hodgkin was a peculiarly unfavorable one, and the great mistake made of not removing the root vessels and filling the canals. He had in mind a case of replantation in tolerably good condition after twelve years' service. From lack of care, and allowing deposits of tartar on one side there was now some absorption on that side.

The whole secret in caring for the teeth is to rub the whole surface of the gums with the finger or tooth brush; there will then be no red margins and no absorption. Another mistake wss in not preparing a proper splint to hold the tooth perfectly quiet from the beginning; it should be held like a broken bone. He had had his failures, it was true, but his successes so far outnumbered them that he felt gratified in urging perseverance in the method. He felt very grateful to Dr. Younger for his experiments in making sockets in edentulous space and resurrecting dead teeth. It strengthens his faith in his own methods.

Prof. Hodgkin said that he was on the mourners' bench, and ready to be converted; especially since he had just learned that his friend Staples, from Texas, had had a central incisor picked up out of the dirt and replaced, which did good service for thirty years.

The subject was passed, and Histology and Microscopy called.

Dr. Stublefield (Nashville, Tenn.,) read a paper on the

"HISTOLOGY OF HARD STRUCTURES."

At the conclusion of the paper, it being nearly midnight, the Association adjourned, to meet on board the steamboat Jane Moseley the next day, *on route* to Washington City.—*Southern Dental Journal.*

